# Formyl peptide receptors in the mucosal immune system

**DOI:** 10.1038/s12276-020-00518-2

**Published:** 2020-10-20

**Authors:** Yu Sun Jeong, Yoe-Sik Bae

**Affiliations:** Department of Biological Sciences, Sungkyunkwan University, Suwon, 16419 Republic of Korea

**Keywords:** Mucosal immunology, Mechanisms of disease

## Abstract

Formyl peptide receptors (FPRs) belong to the G protein-coupled receptor (GPCR) family and are well known as chemotactic receptors and pattern recognition receptors (PRRs) that recognize bacterial and mitochondria-derived formylated peptides. FPRs are also known to detect a wide range of ligands, including host-derived peptides and lipids. FPRs are highly expressed not only in phagocytes such as neutrophils, monocytes, and macrophages but also in nonhematopoietic cells such as epithelial cells and endothelial cells. Mucosal surfaces, including the gastrointestinal tract, the respiratory tract, the oral cavity, the eye, and the reproductive tract, separate the external environment from the host system. In mucosal surfaces, the interaction between the microbiota and host cells needs to be strictly regulated to maintain homeostasis. By sharing the same FPRs, immune cells and epithelial cells may coordinate pathophysiological responses to various stimuli, including microbial molecules derived from the normal flora. Accumulating evidence shows that FPRs play important roles in maintaining mucosal homeostasis. In this review, we summarize the roles of FPRs at mucosal surfaces.

## Formyl peptide receptors: Receptors in the mucosal immune system

Key receptors in defense against bacteria may also be attractive targets for diseases of the mucosa, the tissue that lines and protects areas such as the airways and gastrointestinal tract. Formyl peptide receptors (FPRs) detect substances produced by bacteria and host cells triggering immune or wound-healing responsesYoe-Sik Bae and Yu Sun Jeong at Sungkyunkwan University, Suwon, South Korea, have reviewed how FPRs in the mucosa mediate the immune response. They report that FPRs play many roles, promoting wound closure, enhancing fibrosis of tissues, and promoting or suppressing inflammation in various tissues. FPRs may also be involved in conditions such as Crohn’s disease and asthma. Further study may reveal opportunities for development of FPRs as therapeutic targets in mucosal diseases.

## Introduction

Pattern recognition receptors (PRRs) are mainly expressed in innate immune cells such as neutrophils, monocytes, and macrophages and induce immune responses to infection, injury, or stress by recognizing molecular patterns on antigens. Well-known PRRs include Toll-like receptors (TLRs), nucleotide-binding oligomerization domain-leucin rich repeat-containing receptors, retinoic acid-inducible gene 1-like receptors, and C-type lectin receptors, with TLRs being the most extensively studied among these receptors. Formyl peptide receptors (FPRs), which recognize formylated peptides, are also PRRs^1^. However, the characteristics of this receptor family are different from those of the other PRRs; FPRs recognize various endogenous molecules ranging from mitochondrial formylated peptides to lipid-derived mediators^2^.

There are three types of human FPRs (*FPR1, FPR2, and FPR3*) and eight types of murine FPRs (*Fpr1, Fpr2, Fpr3 (Fpr-rs1)*, and *Fpr-rs3 to 7*). FPR1 was the first receptor discovered in the FPR family; this receptor detects formylated peptides with high affinity. FPR2 has lower affinity for bacterial formylated peptides than does FPR1 but has a wide range of ligands, such as amyloid peptides, antimicrobial peptides, and lipid mediators^2^. FPRs are highly expressed in innate immune cells such as neutrophils, monocytes, and macrophages. However, many reports have also demonstrated that nonhematopoietic cells, including epithelial cells, endothelial cells, neurons, and hepatocytes, express functional FPRs^2^.

Our body maintains its homeostasis and protection from the external environment via different types of barriers. Skin and mucosal surfaces are the barriers separating the inside from the outside of the body^3^. The mucosal surfaces include the gastrointestinal (GI) tract, the respiratory tract, the oral cavity, the urogenital tract, and the eye. Tremendous amounts of commensal bacteria and pathogens exist in mucosal surfaces, and they come in direct contact with the harsh external environment^4^. To defend our body against external attacks, many immune cells are required in the surface region. As a result, in healthy adults, approximately 80% of immune cells are in the mucosal system^5^. Immune responses need to be restricted and regulated at the necessary sites to maintain homeostasis. Immune cells cooperate with other epithelial cells and stromal cells and compose an independent mucosal immune system. The mucosal immune system includes epithelial cells, macrophages, monocytes, neutrophils, B and T cells, innate lymphoid cells, dendritic cells, and natural killer cells^3,6^.

These various components in the mucosal system express PRRs that can recognize normal flora and pathogens. There have been many reports on the role of PRRs in the mucosal system. TLRs are the most extensively investigated PRRs in the mucosal system, and there are many reports on the function of TLRs in the GI tract^7^. TLRs are expressed in intestinal epithelial cells (IECs), and IECs show hyporesponsiveness to ligands of TLR2 and TLR4. Polymorphisms in TLR2 and TLR4 correlate with human inflammatory bowel disease (IBD) pathology^7^. This review summarizes recent reports on the understanding of the expression and function of FPRs in various mucosal surfaces.

## FPRs in the GI tract

The GI tract is also referred to as the digestive tract and constitutes the organ system that connects the mouth to the anus. The GI tract contains the mouth, esophagus, stomach, small intestine, large intestine, and anus. Due to the characteristics of the digestive system, many external stimuli, such as food antigens and bacteria, may affect cellular activities in this region. In particular, the gut of human adults contains over ten times more bacterial cells than human cells. These bacteria build their ecosystem in the gut and maintain homeostasis with epithelial cells and immune cells via mutual interaction. Recently, increased interest in the microbiota has revealed that dysbiosis correlates with various human diseases^4^. In addition, the study of the functional roles of PRRs such as FPRs in the GI tract has attracted interest because many ligands of FPRs, including formylated peptides and lipid metabolites, exist in the GI track.

### FPR1 in the GI tract

FPR1 recognizes formyl peptides with high affinity and is highly expressed in neutrophils. FPR1 is expressed not only in immune cells but also in IECs and intestinal neuronal cells^8–11^. Several reports show that FPR1 is associated with IBD pathogenesis. Crohn’s disease patients have a high expression level of FPR1 in neutrophils^8^, and ulcerative colitis (UC) patients exhibit further activation of FPR1 in their intestines^9^. FPR1 activation in immune cells can induce directional migration of these cells into the inflamed intestinal region. Gliadin, a food antigen that binds to FPR1, decreases intestinal integrity and induces neutrophil migration^12^.

**Fig. 1:**
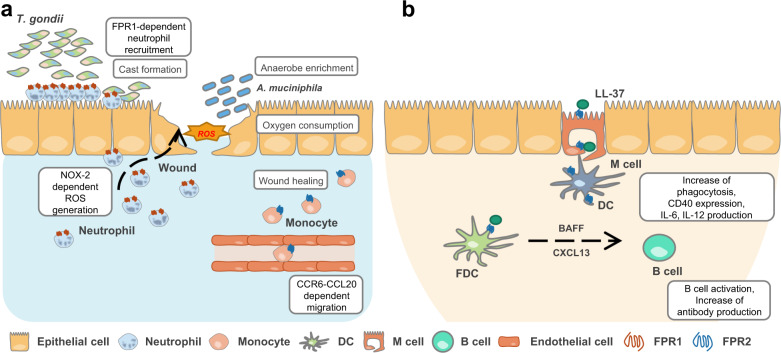
Functional roles of FPRs in the regulation of immune responses in the GI tract. **a** Neutrophils migrate into the inflamed GI tract toward various chemoattractants. In the case of *Toxoplasma gondii* and bacterial infection, neutrophils migrate to the gut lumen in an FPR1-dependent manner to regulate bacterial containment and to separate luminal contents from the epithelium. Neutrophils activated by FPR1 remove microenvironmental oxygen by NOX2-mediated ROS generation, resulting in local enrichment of an anaerobic bacterial consortium. In particular, *Akkermansia muciniphila* facilitates epithelial wound healing through an epithelial NOX1-dependent mechanism. Monocytes, which facilitate epithelial remodeling during wound closure, migrate into the inflamed site via the CCL20-CCR6 axis. FPR2 expression is related to the expression of CCR6 in monocytes. **b** M cells and DCs in Peyer’s patches recognize LL-37 via FPR2, promoting DC activation with increased phagocytosis, expression of CD40, and production of IL-6 and IL-12. Follicular DCs express CXCL13 and B cell-activating factor, supporting germinal center B cell activation in Peyer’s patches via FPR2 signaling.

Neutrophils are the first immune cells that migrate into inflamed tissues, and the most dominant pathological characteristic of IBD is the migration of neutrophils into the intestinal mucosa^13^. Despite the lack of a major chemotactic receptor, neutrophils with *Fpr1* knockout can still migrate into inflamed intestinal mucosa in the dextran sulfate sodium (DSS)-induced colitis model^14^. In DSS-induced colitis, neutrophil migration is induced by other chemokines, such as CXCL2, and FPR1 affects the resolution and recovery of the intestinal barrier. Another report showed that *Fpr1* deletion elicits decreased leukocyte migration into the inflamed intestine in a trinitrobenzene sulfonic acid-induced colitis model^15^. Blocking bacterial dissemination in the intestinal epithelium is a critical factor in regulating the resolution of inflammation. During *Toxoplasma gondii* infection, neutrophils form a ‘cast’ in the gut lumen to separate bacteria from the epithelium and to regulate the bacterial population (Fig. 1a). Neutrophils with *Fpr1* knockout are able to migrate into the lamina propria but cannot migrate into the gut lumen, where they can regulate bacterial containment^16^.

There are some reports demonstrating that introduction of fMLP into the gut induces colitis^17,18^. However, the dose used in these models was extremely high compared to physiological concentrations. Other studies have shown that not only fMLP but also various ligands of FPR1, such as AnxA1, and commensal bacteria, elicit epithelial barrier-protective effects^19–22^. IECs express Hsp27 via FPR1 stimulation, which has an epithelial-protective effect^21^. fMLP decreases TNF-α-induced NF-κB signaling and proinflammatory cytokine production and induces IEC migration. Moreover, fMLP can promote gastric epithelial cell proliferation by interacting with FPR1^22^. FPR1 colocalizes with F-actin and activates Rac1 and Cdc42, which are crucial players in F-actin reorganization in a PI3K-dependent manner^10^.

Reactive oxygen species (ROS) generation is another mechanism of FPR1-mediated wound healing^7^. In immune cells such as phagocytes, FPR1 signaling generates ROS in an NADPH oxidase (NOX)2-dependent manner^2^. On the other hand, ROS generation in epithelial cells via FPR1 activation is mediated by NOX1^23^. AnxA1 promotes wound healing through NOX1-mediated ROS generation in IECs, which inactivates phosphatases such as PTP-PEST and PTEN. These phosphatases have an inhibitory effect on wound healing because they inhibit the activation of FAK and paxillin, which are essential in cell motility and epithelial restitution^19^. Not only AnxA1 but also fMLP and commensal bacteria maintain FAK and ERK phosphorylation and promote wound healing through the same mechanism used by AnxA1, which inhibits the phosphatase DUSP3^24,25^. In addition to epithelial cells, ROS affect the microbiota composition. Neutrophils activated by FPR1 migrate into inflamed mucosa and rapidly remove microenvironmental oxygen via NOX2-mediated ROS generation. As a result, local enrichment of an anaerobic bacterial consortium occurs. *Akkermansia muciniphila*, an anaerobic mucosa-associated bacterium, can also activate FPR1 and induce epithelial cell-specific NOX1-dependent redox signaling^23^ (Fig. 1a).

## FPR2 in the GI tract

FPR2, which has a lower affinity for formylated peptides than FPR1, recognizes diverse ligands^2^. FPR2 is a specific target receptor for various endogenous molecules, such as antimicrobial peptides (AMPs) and specialized pro-resolving mediators (SPMs)^22,26–28^. The expression and distribution of FPR2 are quite similar to those of FPR1, from immune cells such as neutrophils to endothelial and epithelial cells^2^. In the GI tract, FPR2 is expressed on the apical and lateral surfaces of colonic epithelial cells, gastric epithelial cells, and M cells, which are located in Peyer’s patches (PPs)^22,29–31^. The *FPR2* expression level is increased in UC patients compared the healthy controls^32^.

*Fpr2* knockout mice are more susceptible to experimental colitis^33,34^, demonstrating that FPR2 plays protective roles in the GI tract. In the DSS-induced colitis model, *Fpr2/*3 knockout mice showed a more severe disease phenotype and exhibited delayed wound healing due to a decrease in migrating monocytes, which facilitated epithelial remodeling via downregulation of the CCL20-CCR6 axis (Fig. 1a). The CCL20-CCR6 axis is a key chemokine signaling axis that directs migration into the intestinal region. The results of bone marrow chimera experiments indicated that the expression of *Fpr2/*3 in both hematopoietic immune cells and nonhematopoietic cells was essential to protect the intestine in an experimental colitis model^33^. Another result supports the idea that FPR2 plays an essential role in maintaining intestinal homeostasis. The colonic crypt length of *Fpr2* knockout mice is shorter than that of *Fpr1* knockout mice. FPR2 also mediates the wound healing effects of fMLP^34^. Hp(2-20), a bacterial peptide derived from *Helicobacter pylori*, is known to activate FPRL1 (aka FPR2) and FPRL2 (aka FPR3). This peptide exhibits antimicrobial and immunomodulatory activity via FPR activation. Hp(2-20) can induce the migration of gastric epithelial cells via FPRL1 activation and promote the expression of VEGF-A to facilitate wound healing^22^. WKYMVm, which is a synthetic peptide agonist of FPR2, also has a protective effect on DSS-induced colitis. It promotes wound closure and decreases IL-23 and TGF-β production^35^.

Several AMPs can activate FPR2 and show anti-inflammatory effects via receptor activation^26,27,36^. Because the GI tract has the highest microbiota population in the body, the system maintains a high level of AMP production to maintain intestinal homeostasis. The most well-known AMP that binds to FPR2 is LL-37 (human; LL-37, murine; CRAMP). LL-37 is produced by immune cells and epithelial cells and maintains epithelial barrier integrity^37^. It protects the intestine from infection by inducing epithelial cell migration, producing growth factors and mucins, and decreasing epithelial cell apoptosis^38^. LL-37 can also regulate the mucosal immune system as well as epithelial cell activity.

PPs, one of the components of mucosa-associated lymphoid tissue, have the ability to elicit immune responses such as oral tolerance to nonharmful antigens and defense against pathogens. In PPs, several types of immune cells, such as DCs, B cells, and T cells, exist^39^. In particular, M cells and DCs in PPs express FPR2 and recognize LL-37, which results in activation of immune responses. LL-37 conjugated peptide promotes DC activation with increases in CD40 expression, B cell proliferation, and antibody production^30^ (Fig. 1b). Follicular DCs (FDCs) support germinal center B cell activation in PPs via FPR2 signaling. FDCs express CXCL13 and B cell-activating factor, which enhances B cell survival and activation. LL-37 can induce the expression of *Cxcl13*, *Tnfsf13b*, and *Fpr2* in FDCs, resulting in the B cell proliferation and activation and antibody secretion^31^ (Fig. 1b). CSA13, another type of AMP, can ameliorate intestinal fibrosis via FPR2. Intestinal fibrosis is one of the clinically significant symptoms of Crohn’s disease, and CSA13 can decrease fibrosis through FPRL1-dependent inhibition of HMG-CoA reductase activity^40^.

The mucosal system maintains homeostasis by producing SPMs, including lipoxins, resolvins, and prostaglandins^28^. FPR2 is a receptor for lipoxin A4 (LXA4) and resolvin D1 (RvD1) and mediates their intestinal protective effects. UC patients have increased levels of LXA4, AnxA1, and *FPR2*, all of which are components of proresolution pathways^32^. This observation implies that the increased levels of those molecules lead to protective mechanisms to block hyperimmune responses in UC patients. Moreover, LXA4 and FPR2 were found to have protective effects in an ischemia-reperfusion injury model^41,42^. Administration of lipoxygenase inhibitors or FPR2 antagonists was found to be associated with more severe symptoms than administration of the control^41^. This pattern indicates that LXA4-FPR2 signaling is required to maintain intestinal integrity. There are two receptors for LXA4 in the intestinal epithelium: FPR2 and CysLT1. CysLT1 is the receptor for leukotriene D4 and LXA4; however, the protective effect of LXA4 is mediated by FPR2 and not by CysLT1^29^. Endogenous production of LXA4 can be increased by an n-6 fatty acid-enriched diet. In one study, the high n-6 fatty acid-diet group was found to show increased LXA4 levels compared to those in the normal diet group, leading to protective effects against ischemia-reperfusion-induced mucosal injury^42^. RvD1, another SPM, maintains epithelial integrity by inhibiting TNF-α-induced c-Myc expression via FPR2 signaling^43^.

## FPRs in the respiratory tract

The respiratory tract is the organ system involved in the process of respiration. It is divided into the upper respiratory tract and the lower respiratory tract. The upper respiratory tract comprises tissues above the vocal cord, such as the nose, nasal passages, and paranasal sinuses. The lower respiratory tract comprises tissues below the vocal cord, such as the trachea, smaller airways (bronchi and bronchioles), and alveoli. The function of the respiratory tract necessitates its structural feature of a large surface area. Similar to the GI tract, the respiratory tract is in direct contact with various external molecules, including fine particulate matter, bacteria, viruses, and fungi. Maintaining homeostasis between the microbiota and the local immune system is also important in the respiratory tract, as it is in the GI tract^44^. In the respiratory tract, the ligands of FPRs play crucial roles in various inflammatory conditions.

### FPR1 in the respiratory tract

FPR1 is expressed in nasal epithelial cells, lung epithelial cells, lung fibroblasts, and bronchoalveolar lavage fluid (BALF) cells^45–48^. The primary role of FPR1 in the respiratory tract is the promotion of wound healing or leukocyte migration to FPR1 ligands generated upon lung damage. As they can in the GI tract, the ligands of FPR1 can promote wound healing via FPR1 in epithelial cells of the respiratory tract. fMLP and AnxA1 activate alveolar basal epithelial cells and increase the expression of F-actin^49^. Mitochondrial formylated antigens stimulate wound healing in an FPR1-dependent manner. In particular, FPR1 is expressed in the lamellipodia of epithelial cells and directs the migration of these cells during the wound healing process^47^. Lung fibroblasts also migrate and express F-actin via FPR1 signaling. This process occurs through FPR1-mediated, calcium influx, PKC, and PI3K-dependent signaling pathways^48^. Bronchiolitis obliterans syndrome (BOS) is a fibroproliferative disorder caused by transplant rejection. In a murine BOS model, *Fpr1* knockout mice showed attenuation of disease severity with decreased NF-κB nuclear translocation, MAPK signaling, and modulation of the NLRP3 inflammasome. The levels of cytokines directly related to fibrosis, such as VEGF and TGF-β, were also decreased in the *Fpr1* knockout BOS mouse model^50^. These results suggest that FPR1 is detrimental in fibrotic diseases.

**Fig. 2:**
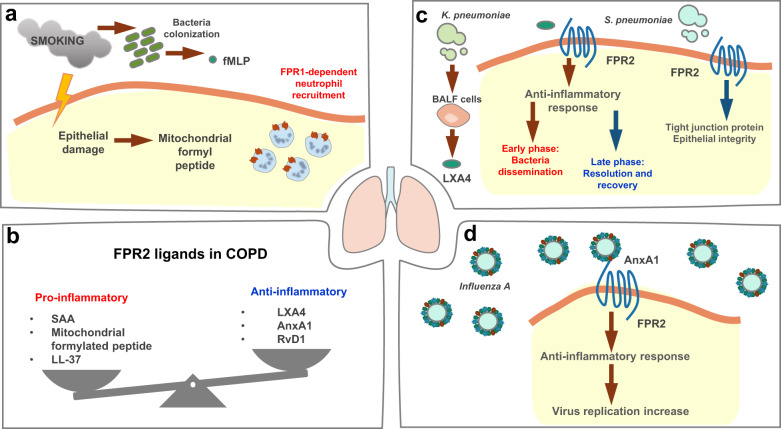
Functional roles of FPRs in the regulation of respiratory diseases. **a** Smoking induces lung damage and structural changes, eliciting augmented bacterial colonization in the lower respiratory tract, with increased production of fMLP. **b** In COPD, inflammatory FPR2 ligands are produced at higher levels than anti-inflammatory FPR ligands. **c** *Klebsiella pneumoniae* infection stimulates BALF cells to produce LXA4, which induces an anti-inflammatory response leading to bacterial dissemination in the early phase of infection and is required for resolution and recovery in the late phase of infection. However, FPR2 is required for maintaining epithelial integrity against *Streptococcus pneumoniae* infection. **d** AnxA1 is incorporated into influenza A virus particles, stimulating FPR2-mediated signaling to block defense activity against virus infection.

Smoking-related lung inflammation is a representative example of the functional roles of FPR1 in respiratory diseases. Smoking is the leading risk factor for chronic obstructive pulmonary disease (COPD) and induces lung damage and structural changes. Bacterial colonization is enhanced in the lower respiratory tract of smokers and causes the production of a large amount of fMLP^51^ (Fig. 2a). The levels of fMLP in BALF and FPR1 expression in neutrophils are higher in smokers than in nonsmokers^52^. The relationship between smoking and emphysema is based on the expression levels of fMLP and FPR1. The expression of FPR1 is more affected by the smoking status than by the COPD status and is restored to the basal level when COPD patients recover to the normal state^53^. *Fpr1* deletion protects against smoking-induced lung emphysema and is linked to decreased leukocyte recruitment and decreased production of inflammatory factors, such as IL-1β, AnxA1, and the anti-inflammatory cytokine IL-10^51^. Smoking cessation cannot stop lung inflammation, because formyl peptide production still occurs. Blockade of FPR1 and FPR2 with antagonists (cyclosporin H or WRW4) can prevent structural deterioration of the lungs^54^. These results suggest that the role of FPR1 in smoking-induced lung inflammation is important in mediating neutrophil migration and lung structural change.

### FPR2 in the respiratory tract

FPR2 is expressed in nasal epithelial cells, bronchial epithelial cells, and BALF cells^55–57^. As FPR2 can detect diverse ligands, many reports have demonstrated the functional roles of FPR2 in the airway. FPR2-related airway diseases and conditions include allergic inflammation, acute lung injury (ALI), lung injury induced by fat embolism syndrome, acute respiratory distress syndrome, ventilator-induced lung injury, asthma, aspirin-exacerbated respiratory disease (AERD), and COPD^55,58–64^. FPR2 can be protective or harmful depending on various disease conditions.

The protective role of FPR2 in respiratory diseases mainly occurs with lipid-derived mediators such as RvD1 and LXA4. RvD1 is a docosahexaenoic acid-derived anti-inflammatory mediator and a ligand of FPR2^65^. In an ALI model, pretreatment with RvD1 was found to attenuate disease onset by decreasing NF-κB nuclear translocation, MAPK signaling, proinflammatory cytokine production, and immune cell recruitment. These beneficial effects of RvD1 on ALI are dependent on FPR2 signaling and are reversed by FPR2 antagonist (BOC-2) administration^65^. In addition to protective effects, RvD1 has therapeutic effects against persistent ALI via the same mechanisms. In acute respiratory distress syndrome, the primary protective mechanism of RvD1 is mediated by increasing the expression of prostaglandin-producing cyclooxygenase 2 in an FPR2-dependent manner^60^.

LXA4, another lipid mediator ligand of FPR2, can decrease TNF-α-induced IL-8 secretion via FPR2 signaling in bronchial epithelial cells^56^. In patients with rhinosinusitis, an upper airway inflammatory disease, the levels of both LXA4 and *FPR2* are increased compared to those in healthy controls, accompanied by augmented defense activity. LXA4 can also decrease TNF-α-induced IL-8 secretion in nasal epithelial cells via FPR2^55^. There are several reports regarding LXA4-FPR2 expression in asthma patients. LXA4 and FPR2 levels are increased in patients with moderate asthma but are significantly decreased in patients with severe asthma. LXA4 decreases leukocyte migration, eotaxin levels, and the production of type 2 cytokines such as IL-5 and IL-13^57,62,66–68^.

Synthetic agonists of FPR2 also protect the airway from inflammation. WKYMVm blocks DC migration to the mucosa and adjacent lymph nodes and inhibits proinflammatory cytokine production, which results in a decrease in Th1 and Th17 responses in asthma^69^. BML-111, another synthetic agonist of FPR2, has a therapeutic effect on ventilator-induced lung injury and lung injury induced by fat embolism syndrome. This compound inhibits leukocyte migration into BALF, production of proinflammatory cytokines, and NF-κB activation^59,61^.

Harmful effects of FPR2 have been reported in several respiratory pathologies, such as allergic inflammation, airway contraction, AERD, COPD, and infection. In the case of allergic inflammation, *Fpr2* knockout mice show attenuated inflammation. The dominant phenotypes of *Fpr2* knockout mice in allergic inflammation are a reduction in DC migration into the mucosa, a decreased in Th2 responses, and immunoglobulin production^70^. In allergic airway inflammation, monocyte-derived DCs are recruited to the perivascular region adjacent to the inflamed area in a CCR2-dependent manner. However, the migration of monocyte-derived DCs into the mucosa is dependent on CRAMP-FPR2 signaling^71^.

Respiratory failure due to lung damage is accompanied by airway contraction, which can be induced by various damage-associated molecular patterns, among which mitochondrial N-formyl peptides act via FPR2^72^. AERD is another disease in which the overactivated LXA4/FPR2 pathway exacerbates the pathogenesis. Because AERD is associated with arachidonic acid metabolism, the role of FPR2 in recognizing LXA4 is important in regulating AERD progression. Single-nucleotide polymorphism analyses have been conducted in AERD patients, showing that these patients have a high frequency of homozygote of the minor allele, *FPR2-4509T*>*G*. This minor allele is correlated with higher FPR2 levels in CD14^+^ monocytes, and carriers show defective lung function upon aspirin challenge^63^.

In COPD, the balance of protective or harmful FPR2 ligands is essential. The protective FPR2 ligands with elevated levels in COPD are AnxA1, LXA4, and RvD1, and the harmful FPR2 ligands are mitochondrial formylated peptides, LL-37, and serum amyloid A (SAA)^64^ (Fig. 2b). The expression of FPR2 is decreased in neutrophils and T cells in COPD patients, and serum AnxA1 levels are decreased^53^. LXA4 treatment can block the proinflammatory response in COPD. However, proinflammatory ligands such as SAA are produced at much higher levels in the disease state, and FPR2-mediated activity therefore leads to inflammatory responses^73^. The primary pathological feature of COPD is neutrophilic inflammation. However, glucocorticosteroids, which are currently used as therapeutic agents for COPD, fail to alleviate neutrophilic inflammation, because glucocorticosteroids induce SAA production^64^. The levels of SAA in serum and BALF are high in COPD patients, and the environment of epithelial cells is rich in SAA^73^. SAA induces the expression of *Il8* and *Cxcl1/2* and the production of proinflammatory cytokines in BALF cells. Among these cytokines, IL-17A, produced by Th17 cells, γδ T cells, and epithelial cells, maintains pulmonary neutrophilic inflammation^74^. LL-37 is an FPR2 ligand that plays a deleterious role in COPD. LL-37 is highly expressed in the epithelium of COPD patients, and the expression level of LL-37 positively correlates with lung structural changes such as airway wall thickness and collagen deposition. The harmful effects of LL-37 are mediated by FPR2 activation in human lung fibroblasts, promoting the production of collagen^75^.

FPR2 has dual effects on airway infection. Regarding bacterial infection, *Fpr2/3* knockout mice and *AnxA1* knockout mice are more susceptible to *Streptococcus pneumoniae* infection than wild-type mice. These knockout mice show decreased levels of tight junction proteins (ZO-1 and claudins), disrupted macrophage phagocytosis and uncontrolled inflammation, resulting in pulmonary dysfunction^76^. The results suggest that FPR2/3 are crucial for *Streptococcus pneumoniae* defense. However, the opposite functional activity of FPR was reported in the *Klebsiella pneumoniae* infection-induced sepsis model. In the early stage of pneumosepsis, the expression of LXA4 and FPR2 is elevated and causes defective bacterial clearance. FPR2 antagonist treatment reverses the effect of LXA4/FPR2 by inducing leukocyte migration into the infection site and increasing bacterial killing. However, in the late stage of pneumosepsis, LXA4/FPR2 participate in the resolution of inflammation, influencing mortality^77^ (Fig. 2c). Maintaining the balance between benefit and harm is important for an effective response to bacterial infection. Viruses often manipulate FPR2 signaling to escape immune clearance by exploiting its anti-inflammatory properties. Influenza A virus (IAV) is an example of a virus that uses this strategy. TLR3 expressed in innate immune cells and other cell types recognizes IAV and induces the production of type 1 interferons. The produced interferons activate STAT3, which increases the transcription of FPR2. Upregulated FPR2 carries out its anti-inflammatory functions and subsequently facilitates viral replication^78^. AnxA1 is incorporated into IAV particles, eliciting the activity of the FPR2-ERK-dependent pathway to play a deleterious role in host defense activity against IAV infection^79^ (Fig. 2d).

## FPRs in other mucosal surfaces

### FPRs in the oral cavity

The oral cavity is the first site at which external substances enter before proceeding into the GI tract and respiratory tract. Many kinds of stimulants, such as food, allergens, and pathogens, enter the oral cavity. The oral cavity is the site with the second largest microbiota after the large intestine^4^. Because the fundamental role of the mouth is eating, there is constant mastication and physical stimulation. The oral cavity comprises several barrier surfaces, including the lining mucosa, masticatory mucosa, tongue mucosa, and gingival crevice. These surfaces are kept moist by saliva and gingival crevicular fluid^80^. The immunological feature of the oral cavity is that both mucosal immunity (soluble IgA) and systemic immunity (plasma IgG) protect this site^81^. Because of the complex immunological conditions, all immune cell types are resident and active in the oral cavity^80^.

Reports on FPRs in the oral cavity have focused on very narrow aspects: reports on FPR1 have focused on periodontitis, and reports on FPR2 have focused on submandibular gland and saliva production. Periodontitis is a disease caused by gingival infection. Since the 1970s, it has been well known that neutrophils from periodontitis patients have defective FPR1 signaling^82,83^. In particular, neutrophils from juvenile periodontitis patients showed decreased migratory ability towards fMLP but not C5a^83^. From these findings, researchers uncovered genetic variations in the *FPR1* gene in periodontitis patients^84–88^. Although the type of periodontitis was different, the genetic variations in *FPR1* from periodontitis patients had a common feature; the mutations caused the receptor to malfunction. The *FPR1-329T*>*C* and *FPR1-378C*>*G* variants cause F110S and C126W amino acid sequence alterations, respectively^84^. These mutations are associated with decreased migratory ability towards fMLP and defects in G protein coupling^85,86^. In particular, mutation of amino acid 110 affects the surface expression of FPR1^86^. Other mutations that occur are R190W and N192K, which change the extracellular loop structure of FPR1 and decrease its ligand binding affinity^87^.

Studies on the role of FPR2 have focused on the submandibular gland. The submandibular gland is one of the major salivary glands located under the floor of the mouth. Saliva is a watery fluid produced from salivary glands that contains various innate antimicrobial molecules, such as immunoglobulins, lysozyme, and lactoferrin. Decreased saliva production or the absence of saliva increases susceptibility to oral infection^80^. FPR2 and its ligand RvD1 reduce immune responses in the salivary glands. *Fpr2* knockout mice were found to show an enhanced immune response in the salivary gland in an LPS challenge model. These knockout mice exhibited abnormalities such as increased immune cell infiltration in the salivary gland, upregulation of inflammatory cytokines, decreased saliva production, increased apoptosis, and alterations in tight junction proteins^89^. RvD1 activates FPR2 on the salivary glands and helps to maintain salivary gland integrity and to inhibit apoptosis and TNF-α-mediated inflammation^90,91^.

Sjogren syndrome (SS) is a chronic autoimmune disease that decreases the function of exocrine glands. As a result, the production of tears and saliva decreases. One study focused on the effect of RvD1 on NOD/ShiLtJ mice, whose phenotype mimics human SS^92^. RvD1 activates FPR2 expressed on submandibular gland cells, showing a preventive effect on the onset of disease by improving secretory function, decreasing proinflammatory molecule expression, and increasing anti-inflammatory molecule expression and M2 macrophage polarization. Interestingly, old female *Fpr2* knockout mice show symptoms similar to those of SS, such as reduced salivary flow and weight loss^93^. *Fpr2* knockout mice have an increased CD20^+^ B cell population and enhanced autoantibody production in the submandibular glands. This observation implies that FPR2 maintains the functional status of salivary glands by shaping adaptive immunity and regulating the inflammatory response intensity.

### FPRs in the eye

The eye is an organ of the visual system. The structure of the eye evolved anatomically and immunologically to protect visual function^81^. Significantly, the exterior surface of the eye is in direct contact with the external environment and encounters an enormous amount of immunogens. The immune response in the eye is strictly regulated to maintain the transparency of the cornea. The ocular mucosal surface includes the cornea and the conjunctiva. Tear film protects these mucosal surfaces, and neural inputs from the cornea and conjunctiva regulate the production of tears. Tears contain various protective substances, such as mucin produced by goblet cells and immunoglobulins produced by plasma cells^81^.

FPRs are expressed on various cell types in the ocular region, such as corneal endothelial cells, corneal epithelial cells, conjunctival goblet cells, retinal microglia, retinal pigment cells, and lens epithelial cells^94–99^. Additionally, various FPR-related ocular pathologies exist, such as retinal degeneration, uveitis, polypoidal choroidal vasculopathy, corneal neovascularization, and ocular allergy^97,100–103^. However, here, we only discuss the role of FPRs in the cornea and conjunctiva as ocular mucosal surfaces. The cornea is located at the anterior aspect of the eye and passes light to the lens. The immune response in the cornea is unreactive compared to that in the conjunctiva, because inflammation of the cornea can affect its transparency^104^. FPR2 is expressed on the corneal endothelium and epithelium, and the functional activity of FPR2 in those regions has been indirectly confirmed by effects of the FPR2 antagonist WRW4^94,95^. The cornea is a transplantable tissue, and there are several reports that the ligands of FPR2, including LXA4 and RvD1, can be beneficial during corneal transplantation. LXA4 promotes the proliferation of human corneal endothelial cells, and when added to Optisol-GS, which is the pretransplant storage fluid for corneas, LXA4 reduces corneal endothelial cell damage^94^. RvD1 reduces allograft mortality during corneal transplantation by regulating DC-mediated inflammatory responses^105^. Although RvD1 promotes corneal wound healing, it is not clear whether FPR2 mediates the effect of RvD1 in vivo.

Diabetes is a representative metabolic disease with ocular complications. Diabetic patients show impaired corneal wound healing because of corneal epithelium and nerve defects. RvD1 can improve corneal epithelial regeneration in diabetic mice. RvD1 acts via the following mechanisms: increased expression of proliferation-related molecules (Ki67 and SIRT1), EGFR activation, decreased inflammatory cytokine (TNF-α, IL-1β) production, decreased expression of ROS-generating enzymes (NOX2 and NOX3), and upregulated expression of antioxidant genes (Nrf2, MnSOD, and HO-1). WRW4, the FPR2 antagonist, significantly blocks these various therapeutic effects of RvD1^106^. Another FPR2 ligand, LL-37, also promotes corneal epithelial cell migration in a PTX- and FPR2-sensitive manner and facilitates corneal wound healing^95^. Regarding SAA, the expression of *Saa1,3* was increased with the expression of *Fpr2* in a corneal neovascularization mouse model^102^. This pattern implies that SAA-FPR2 plays a role in this condition.

The conjunctiva is a mucosal tissue composed of three layers: the conjunctival epithelial layer, goblet cell layer, and lamina propria. The lamina propria is the site where immune cells reside and produces immunoregulatory factors. In the conjunctiva, several immune responses, such as antigen processing, cell-mediated immunity, and hypersensitivity, occur^104^. The conjunctiva exhibits basal expression of FPR1 and FPR2, and FPR1 and FPR2 expression is elevated in the setting of inflammation, such as in allergic conditions^103^. FPR2 is expressed on conjunctival goblet cells and increases mucin production by recognizing LXA4 and RvD1^96,107^. Intracellular calcium influx is directly related to glycoconjugate secretion, and the LXA4-FPR2 axis can promote mucin secretion via calcium influx^96^. Various downstream signaling molecules of FPR2 affect calcium influx, which shows that FPR2 signaling and homeostasis of the ocular environment are closely related^107^.

One report addressed AnxA1 and FPRs in the case of ocular allergy. Upon the induction of allergic conjunctivitis in mice, FPR1 and FPR2 expression was increased. Interestingly, *AnxA1* knockout mice showed a significant increase in *FPR2* expression. AnxA1 is an anti-inflammatory mediator that decreases granulocyte infiltration and proinflammatory cytokine production in allergic conjunctivitis. These protective effects were diminished by treatment with Boc2, a pan-FPR antagonist^103^.

## Conclusion

We summarized the roles of FPRs in the mucosal system. In the mucosal area, several different cells express functional FPRs. FPRs have dual effects depending on their ligands expressed under various pathophysiological conditions. Epithelial cells promote wound closure via FPR-dependent signals in the GI tract and respiratory tract. FPR1 exacerbates fibrosis by activating fibroblasts, and FPR2 alleviates fibrosis by regulating HMG-CoA reductase activity. In immune cells, as chemoattractant receptors, FPRs recruit leukocytes to sites of inflammation or mucosal effector sites. Extensive research about FPRs in other mucosal surfaces, such as the oral cavity and the eye, has not been conducted. However, it is clear that FPRs contribute to maintaining tissue homeostasis at these sites. Since FPRs play diverse roles as chemoattractant GPCRs and PRRs, they have great promise as targets for the development of therapeutic agents to control mucosa-related diseases.
